# Quotas: Enabling Conscientious Objection to Coexist with Abortion Access

**DOI:** 10.1007/s10728-020-00419-5

**Published:** 2020-11-19

**Authors:** Daniel Rodger, Bruce P. Blackshaw

**Affiliations:** 1 School of Health and Social Care, Allied Health Sciences, London South Bank University, 103 Borough Rd, London, SE1 0AA UK; 2 Department of Philosophy, University of Birmingham, Birmingham, B15 2TT UK

**Keywords:** Conscientious objection, abortion, rights, quotas, professional obligations, pregnancy

## Abstract

The debate regarding the role of conscientious objection in healthcare has been protracted, with increasing demands for curbs on conscientious objection. There is a growing body of evidence that indicates that in some cases, high rates of conscientious objection can affect access to legal medical services such as abortion—a major concern of critics of conscientious objection. Moreover, few solutions have been put forward that aim to satisfy both this concern and that of defenders of conscientious objection—being expected to participate in the provision of services that compromise their moral integrity. Here we attempt to bring some resolution to the debate by proposing a pragmatic, long-term solution offering what we believe to be an acceptable compromise—a quota system for medical trainees in specialties where a conscientious objection can be exercised, and is known to cause conflict. We envisage two main objectives of the quota system we propose. First, as a means to introduce conscientious objection into countries where this is not presently permitted. Second, to minimise or eliminate the effects of high rates of conscientious objection in countries such as Italy, where access to legal abortion provision can be negatively affected.

## Introduction

The protracted debate regarding the role of conscientious objection in healthcare has recently intensified [5, 10, 21–23, 36, 45, 51], and there have been few practical solutions suggested likely to satisfy both opponents and proponents of conscientious objection. One of the primary concerns of opponents to conscientious objection is ensuring that eligible patients have access to legal medical services that they need or request. They see conscientious objection as an obstacle to this, and consequently believe the right to conscientious objection should be removed [19, 50]. Conversely, conscientious objectors typically have deeply held moral beliefs regarding certain services such as abortion, and do not wish to participate in their provision. Of course, supporters of a *right* to conscientious objection are not necessarily objectors themselves, but they are still concerned that those who do wish to object are able to do so.

Our contention is that in a liberal society, the right of healthcare professionals to conscientiously object to their involvement in the provision of certain healthcare services such as abortion is of considerable value, both to themselves, and to the healthcare system in general. However, if the proportion of objectors in the healthcare system is sufficiently high, the provision of these legal services could be compromised. We believe it is important that both of these competing concerns should be accommodated. If a viable solution does not emerge, it seems likely to us that the debate will grow increasingly entrenched, and current demands for curtailing or removal of existing conscientious objection freedoms are likely to grow stronger. Therefore, we have developed a pragmatic long-term proposal for a conscientious objection quota system that we believe can, to a large extent, minimise these concerns. It is intended primarily for use in two different contexts.

First, where the effect of conscientious objection is raising what seem to be genuine concerns regarding service provision. For instance, in Italy it has been argued that the high proportion of healthcare professionals who are conscientious objectors to abortion is in some cases, hindering access to abortion. If this impact can be convincingly demonstrated, the introduction of quotas would serve to limit the number of medical trainees with a conscientious objection to involvement in the provision of abortion services in specialties such as obstetrics, gynaecology, and general practice.^1^

Second, in countries where conscientious objection is not currently permitted, quotas could be used to help open up certain specialties to those whose moral beliefs currently mean they are unable to consider these areas for potential employment. Importantly, for critics of conscientious objection, this would come with little risk of compromising access to those services if the quota remained low and was carefully regulated. One rationale for the introduction of conscientious objection in this context would be to alleviate staff shortages without compromising patient care. Another is to accrue the more general benefits of permitting conscientious objection that we will outline. We believe this is a novel proposal that has the potential to resolve much of the current deadlock, satisfying the concerns of the majority of those involved in the debate. Importantly, our proposal provides a path in these countries to bring individuals into specialities and professions such as obstetrics and midwifery, who at present feel they have no place in that profession because of their moral beliefs and their inability to conscientiously object.

In countries where conscientious objection is permitted but is not currently known to be compromising access to abortion services, our proposal will also serve to moderate calls for its removal, by providing a viable alternative should it be required. However, we believe the implementation of quotas must be predicated on robust evidence that shows that legal access to abortion services *is* being impacted by high rates of conscientious objection.

## State of the Debate

The volume of philosophical and medical discourse on conscientious objection has increased rapidly in the last few years, [55] and grown increasingly intractable, particularly regarding abortion provision. Certain ethicists have strongly rejected any accommodation of conscientious objection. For example, Christian Fiala and Joyce H. Arthur have described conscientious objection as ‘dishonourable disobedience’ and ‘an abandonment of professional obligations to patients’ [19]. Along similar lines, Udo Schuklenk and Ricardo Smalling assert that ‘medical professionals have no moral claim in liberal democratic societies to the accommodation of their individual conscientious objections’ [54]. In the context of assisted dying Schuklenk [53] espouses what Mark Wicclair calls the ‘incompatibility thesis’, [60] whereby conscientious objection is understood to be incompatible with professional obligations.

One of their main stated concerns is that the right to conscientious objection may limit access to certain medical services [19, 54]. Another is the suspicion that some conscientious objection claims are not genuine but are used to avoid any perceived stigma or have other motivations rooted in self-interest [34]. Conversely, those in favour of conscientious objection typically have moral beliefs regarding certain procedures such as abortion—euthanasia and assisted dying—and do not wish to compromise their consciences by being required to participate [6, 7].

There have been numerous attempts to develop criteria for determining what conscientious objections should be permitted. Rawlsian public reason has been proposed as one possible path: Robert McConnell and Robert Card’s public justification approach requires reasons to be presented for evaluation according to a comprehensive set of criteria [42]. The use of medical tribunals has also been put forward to distinguish between justified and unjustified conscientious objections [32].

Savulescu and Schuklenk believe that conscientious objections should not be permitted for procedures within the scope of professional practice [51]. Toni Saad and Gregory Jackson argue that practices should conform to the norms of medicine, chief of which is the restoration of health. Medical practices that are not aimed at the restoration of health can therefore legitimately be objected to [49]. Others have argued that any acceptable conscientious objection must balance the patient’s interests and the preservation of the health professional's moral integrity [40]. David Oderberg has suggested objections may qualify if there is a ‘history of dispute between recognised bodies of thought or over which reasonable people have disagreed or could disagree’ [47].

The difficulty with these approaches is that they do not address the primary concerns of all the involved parties. Often enough, those who are opposed to conscientious objection do not wish to see *any* procedures denied, irrespective of criteria that *might* limit the scope of objections. Some are suspicious that individuals may lie or exaggerate their beliefs [19] or base their objections on idiosyncratic or discriminatory beliefs [50, 62]. These concerns are not unfounded—for instance, there have been reports of doctors refusing to provide abortion services on grounds other than legitimate conscientious objection [37], such as because of stigma or financial implications for their private practice [13].

For those who conscientiously object to involvement in abortion provision, the prospect of having to meet certain criteria or publicly justify their objections is concerning. If their objection does not qualify, they are placed in the difficult position of having to comply or submitting to whatever penalty is mandated. Ultimately, they may have to leave their position.

We believe a compromise is required. Ideally, opponents to conscientious objection need to agree that healthcare professionals should not be required to participate in or provide services that they strongly believe to be morally objectionable—not a major concession as long as service provision is unaffected, except perhaps for the most strident opponents. However, advocates of conscientious objection also need to agree that the practice should not prevent patients from receiving services they are legally entitled to receive. If it can be shown that provision is being compromised, they need to be willing to accept *some* restrictions to alleviate this. If their right to object is to be maintained, then there needs to be a path to reducing the number of objectors to levels where provision is no longer affected. We believe our quota system meets these requirements. Before we explain the details, we will first examine the current impact of conscientious objection on abortion services—one of the primary concerns of opponents of conscientious objection.

## Is Conscientious Objection Affecting Abortion Provision?

At least 22 countries in Europe permit conscientious objection through regulation [8], and in those with low prevalence of conscientious objection there is little evidence that the provision of abortion services are being compromised. In England, Portugal, and Norway where abortion is legal, it is generally accepted that access to legal abortion is not compromised by permitting individual clinicians to exercise a legal right to conscientious objection [8]. However, accurate data on the actual number of conscientious objectors in many countries remains scant and remains an area requiring further research. A survey of 131 obstetrics and gynaecology trainees from the United Kingdom (UK) in 1998 showed that 31% were conscientious objectors to abortion in the first trimester, although 88% of the trainees would participate in an abortion for fetal abnormality [48]. This adds an additional complication, because in some cases a self-identified conscientious objector may still provide some abortion services.

A more recent study from Ghana provided a more detailed picture of the prevalence of conscientious objection from a sample of 213 healthcare practitioners [3]. The study found that 37.9% of the trained abortion providers self-identified as conscientious objectors. Interestingly, very few of the study’s participants—physicians, midwives, nurses, and physician assistants—wanted to penalise conscientious objectors, although a significant proportion of respondents supported increased regulation that included mandatory confidential registration of objectors [3]. In a cross-sectional survey of midwives in Ethiopia, over 25% of the 188 sampled were unwilling to provide abortion services [30].

The situation in Italy is frequently cited as an example of where a high prevalance of conscientious objection is compromising abortion provision [43]. Data from the Italian Ministry of Health shows that close to 70% of Italian gynaecologists and over 50% of anaesthetists exercised their right to conscientious object to abortion, as well as a significant percentage of non-medical staff, and this proportion has increased over the last decade. Furthermore, in Italy, only 60% of hospitals with gynaecology and obstetrics services provided abortions, and it has been stated that this is due to the normalisation of conscientious objection [8]. The circumstances in Italy led to the International Planned Parenthood Federation in 2012 and the Italian General Confederation of Labour in 2013 to file claims with the European Committee for Social Rights (ECSR) [15, 16]. In both cases the ECSR ruled that Italian women can experience barriers and discrimination when seeking access to abortion services and that the measures to ensure access are currently inadequate.

The Italian government disputed these conclusions on empirical grounds and has stated that they cannot limit the number of medical personnel whilst respecting freedom of conscience without contravening Article 9 of the European Convention on Human Rights [17]. The Italian Committee for Bioethics subsequently argued that despite the high numbers of conscientious objectors the available data does not show that abortion provision is negatively affected [33]. For instance, in some Italian regions, the number of conscientious objectors increases while waiting times for abortions decrease. Conversely, in other regions the number of objectors decreases and waiting times increase. Bo et al. have responded by arguing that the committee’s approach is methodologically flawed, and that the data is insufficient to exclude a causal relationship between the number of conscientious objectors and waiting times, and that more research is required [5]. However, more recent evidence has shown that the high rates of conscientious objection in Italy can compromise access to abortion provision at the local level—affecting waiting times, travel distances, and requiring many women in Italy to obtain an abortion in a different region [1].^2^ To summarise, there is evidence for a link between conscientious objection and compromised access to abortion services, but it is limited to areas where levels of conscientious objection are very high.

## Conscientious Objection Trends

Pressure is being exerted from various quarters with the curtailment of conscientious objection as a goal. One example can be found in a 2018 report—produced by an expert group of 45 activists, health professionals, academics, and lawyers from 22 countries—called ‘Unconscionable: When Providers Deny Abortion Care’ [59]. The contributors argue that the term ‘conscientious objection’ is a misnomer that is more accurately characterised as a ‘refusal to provide services’ and should not be permitted [59]. There are several European countries such as Sweden, Iceland, Finland, the Czech Republic and Bulgaria which do not permit conscientious objection, and are often used as exemplars to demonstrate that conscientious objection is unnecessary [19, 26, 44, 59].

More concerning for supporters of the right to conscientiously object are the laws being passed in countries that have recently decriminalised abortion. The Australian states of Queensland and New South Wales have recently done so, as has Ireland and New Zealand. In all these cases, conscientious objectors are legally required to refer patients to another healthcare professional. This is problematic for objectors, because as Thomas Finegan has pointed out, referral is still material cooperation: ‘the obvious, foreseen side effect of her making the referral is that her patient’s plan to abort is aided in a significant way’ [20]. In the United Kingdom, a 2014 Supreme Court judgement defined participation in a narrow sense, so that medical staff working in ancillary or administrative roles who object to abortion must still arrange abortions. The judgement also stated that the conscientious objector is ‘under an obligation to refer the case to a professional who does not share that objection’ [25]. During 2014, the Norwegian government attempted to strengthen conscientious objection rights by permitting GPs to refuse referrals. Popular protest ensured that the changes were withdrawn [8].

Similar issues are being encountered in countries which are legalising euthanasia and assisted suicide. Typically, conscientious objection provision is written into the laws or regulations for a specific procedure such as abortion, giving an opportunity for different conscientious provisions to be employed for euthanasia. For example, in Canada, the 2016 law regarding medical assistance in dying does not mention conscientious objection. Physicians are not compelled by this law to be involved, but they may be required to do so by other regulations. For example, the College of Physicians and Surgeons of Ontario dictates that physicians must provide the patient with an effective referral [9]. Similarly, in Maine in the United States, the recent Maine Death with Dignity Act obliges physicians to provide patients with information regarding assisted suicide, and ‘do everything preparatory to the actual prescription of lethal medication’ [41].

Thus, the primary way conscientious objection rights are being eroded in various jurisdictions is when new legislation is enacted to legalise what was previously an illegal practice—usually abortion, euthanasia and assisted dying. Because conscientious objection provisions are tied to specific procedures, when this occurs there is a new opportunity to restrict the exercise of conscience. This is not ubiquitous—for example, in Victoria, Australia, the conscientious objection provisions in the Voluntary Assisted Dying Act 2017 are strong. The Victorian law is, however, very much the exception in a growing trend.

## Why Keep Conscientious Objection?

Given the trend towards gradually curtailing the right to conscientiously object in numerous countries, a question worth posing is why conscientious objection should be maintained at all. If it does have the potential to compromise access to legal services such as abortion, then the simplest solution would seem to be to eliminate conscientious objection altogether. The numerous commentators we have cited who describe conscientious objection in disparaging terms [19, 53] certainly imply this is their preference—and some explicitly call for its removal altogether [18, 51]. Doing so would avoid any economic investment and the resources required to establish the quota system that we propose. As an anonymous reviewer suggests, it may be that the resources required for these activities could be better spent on actual healthcare provision. Similarly, Schuklenk and Smalling state that there is no reason that ‘any healthcare system should burden itself, and ultimately patients, with these sorts of logistical problems when there is an obvious, more efficient alternative: saying no to the conscience-based accommodation requests of healthcare professionals’ [54].

There are, however, several reasons why permitting conscientious objection is desirable. Firstly, as Mark Wicclair states, if a healthcare practitioner is forced to provide services contrary to their core moral beliefs, their moral integrity is compromised—and our moral integrity can be extremely important to us [61]. Critics may reply that if performing certain legal medical services violates a practitioner’s moral beliefs, then they should choose a different profession, but this fails to acknowledge that the legality of certain procedures may change during the course of someone’s career. For example, in 2007 Mexico City decriminalized abortion, and Ireland did so in 2018. Requiring all healthcare professionals who object to involvement in abortion services to leave the profession once it is decriminalised seems unfair if there was no possibility of their involvement when they first trained for their profession.

Secondly, an uncompromising position on conscientious objection may result in staff shortages by discouraging entry into certain healthcare professions. For example, Sweden does not permit conscientious objection, despite having a chronic shortage of midwives for several years [28, 29]. In some cases, women have had to travel up to 200 km to give birth due to the closure of several maternity units [31].^3^ In 2017 two Swedish midwives filed complaints with the European Court of Human Rights in Strasbourg after being denied employment to practice there. This resulted in one of the midwives commuting to Norway where she was free to work and exercise a right to conscientious objection [52]. It is worth noting that abortion provision constitutes very little of the work of a midwife in Sweden—one study has shown that 35% of midwives had never provided any abortion care [38].

Finally, Morten Magelssen argues that moral integrity is part of the common good and is a particularly important quality for healthcare professionals. He points to past atrocities in medical history which might have been prevented if those involved had been more willing to object [40]. We have previously described how even critics such as Savulescu and Schuklenk concede there are situations where healthcare professionals must act on their conscience [4]. It is also conceivable that in the future, some practices that are currently legal may come to be regarded negatively and eventually be banned, and conscientious objection may be an important part of that process.^4^ Holly Fernandez Lynch adds that prohibiting conscientious objection is likely to reduce diversity in the healthcare profession, which is valuable to patients [39]. She also suggests that if the right to object on moral grounds is prohibited, this may contribute to public distrust of the healthcare profession [39].

Complete removal of conscientious objection, therefore, may harm individual healthcare professionals, result in staff shortages in some areas, and make it more likely that practices that are subsequently judged to be unethical are tolerated by the healthcare community.

## Introducing Quotas

Given the benefits of permitting conscientious objection, there seems to be no justification for its removal in countries where it has no discernible impact on abortion provision, despite the demands of some critics [18, 51]. However, in countries where there is clear evidence that abortion provision is being affected by high levels of conscientious objection, some changes are obviously required if such services are to be available to all. In these circumstances, it seems pragmatic for both sides to compromise—to endeavour to preserve the right to conscientiously object, but to address the primary concerns of critics regarding service provision.

Francesca Minerva outlines three practical suggestions to address the state of conscientious objection and abortion in Italy [43]. First, she suggests changing Italian law to allow general practitioners to perform early term abortions, as currently only gynaecologists and obstetricians can, thereby significantly increasing the number of willing participants. Second, financial incentives or additional annual leave should be provided to non-conscientious objectors. Third, hospitals should be required to maintain a sufficient ratio of conscientious objectors and non-conscientious objectors to ensure that abortion services are not compromised. Minerva suggests that each hospital maintain a 50/50 split until empirical studies have been conducted to demonstrate an ideal ratio. If a non-conscientious objector later becomes a conscientious objector, this may require terminating their employment.

We think Minerva’s suggestion of maintaining a certain ratio of objectors to non-objectors is a step in the right direction. One concern is that in Italy, where we have noted that a significant proportion of clinicians are conscientious objectors, implementing a 50/50 split would entail many losing their positions, even though up to the time of implementation they would have been legally entitled to object. Again, this seems unfair, and the job market will likely remain saturated with clinicians who are conscientious objectors. Additionally, imposing quotas on clinicians in such a manner does not address the source of the problem—that, potentially, too many of those entering a speciality may wish to conscientiously object. Consequently, our preference is to intervene earlier, at the point at which clinicians choose their speciality.

Provided parties to the debate can agree to respect their opponents’ positions in this regard, we argue there is a practical and realistic approach that addresses the primary concerns of all concerned: the use of a *quota system* for specialist medical trainees (or other relevant professionals such as midwives) as a means to regulate the proportion of professionals that are permitted to conscientiously object. We believe such quotas would address the conflict between a woman's legal right to access abortion services and the right of healthcare professionals to conscientiously object to providing these services.

The basic concept is to reserve a specific proportion of ‘conscientious objection’ places as part of an annual intake to a specialist training pathway. Once this proportion is met, no more trainees with a conscientious objection would be able to enter that specialist training pathway until a subsequent intake. The quota of conscientious objection places does not need to be met in each intake; rather, it functions as a maximum to preserve access to legal service provision. This ensures that trainees and later consultants maintain their legal right to conscientiously object whilst minimising—although not completely removing—the risk of legal service provision being compromised.

The advantages of such a system could be significant. The percentage of conscientious objection places could be set appropriately to prevent there being a shortage of clinicians willing to provide legal services that are commonly objected to, addressing one of the major concerns of critics. It would also permit an agreed proportion of clinicians to practice with very little risk of being required or expected to compromise their moral beliefs regarding services such as abortion—or having to justify their objections to a committee or tribunal. A quota also reduces the theoretical risk of having a medical specialty constituted entirely of conscientious objectors.

A quota system could also be used as a conservative approach to introduce conscientious objection rights to the countries we noted above that currently do not permit it. If set to low levels, there should be no impact on service provision, and yet it would serve to open up certain medical specialties or professions to those who were unable to commit to careers in these fields because of their moral beliefs. We have already noted Sweden’s shortage of midwives. The use of a quota for access to midwifery training in Sweden may help to relieve the shortfall in midwives to some extent, with very little risk of affecting access to abortion services. This is likely to result in a net improvement in care at childbirth without affecting abortion access, and would seem to justify the introduction of such a quota system.

## Determining Quotas

One of the most crucial components of the proposed quota system is deciding what healthcare practices warrant the introduction of a quota, and at what percentage they should be set. The most obvious candidate—and our primary focus here—would be specialties and professions involved in the provision of abortion because of its wide legal availability and the ethical debate that surrounds the practice.^5^ These proposals are tentative, and are primarily meant to initiate a conversation around the concept of training quotas.

There are two scenarios we have proposed as being suitable for the introduction of quotas. The first is countries where conscientious objection has been shown to have an impact on the provision of abortion services, such as Italy. The second is countries that currently do not permit conscientious objection, and where it is thought its introduction would be beneficial in some way, such as helping to alleviate staffing pressures. Doing so should increase the pool of applicants to certain specialist training pathways and some healthcare professions such as midwifery and nursing.

Keeping in mind that quotas on specialist training places will take some time to have an impact on service provision, how might they be calculated in a case such as Italy? We have noted that almost 70% of Italian gynaecologists are conscientious objectors, so clearly—all else being equal—a lower figure is required for a training quota, especially as our proposal does not impact existing practitioners. Surveying existing trainees may suggest a figure—if, say, only 50% of existing trainees report that they would be conscientious objectors, then perhaps 50% would be a suitable quota. The key would be to enforce a quota that is significantly less than the existing percentage of conscientious objectors that are affecting service provision. In time, this will result in a lower percentage of overall conscientious objectors. There will be a temptation to temporarily introduce quotas that are extremely low or even zero for a short period to more rapidly alleviate service provision difficulties, but this could well be counter-productive—in a society with high levels of conscientious objection, it may result in a steep fall in applications to specialise in obstetrics and gynaecology. This could then impact maternal care more generally in the future if there were fewer qualified obstetricians and gynaecologists.

Implementation of quotas would be quite different for a country that currently does not permit conscientious objection—for those in favour of its introduction, any quota would be an improvement. Experiences of other countries that do not experience difficulties with service provision could be used as a guide as to what levels of conscientious objection do not have adverse effects. Again, as training quotas have little effect in the short-term, quotas could initially be generous enough that all entrants who wish to register as conscientious objectors could be permitted to do so—this might be a way of rapidly increasing the number of qualified practitioners if there is an overall shortage in a particularly speciality or profession. Once the effects of this can be measured, a lower quota could eventually be introduced to prevent difficulties in service provision from arising in the future.

An important corollary to the introduction of a quota system should be to gather detailed and accurate data regarding conscientious objection, so that an evidence base of its prevalence and effects can be gathered over time. This should provide a clearer picture of how conscientious objection is affecting service provision under the quotas that have been set. If the effect is minimal or non-existent, then it seems reasonable to grant that the current quotas could remain.

Let us now examine possible objections to our proposed quota system, including some practical implementation issues. We conclude that none are insurmountable.

## Imposition of Constraints

Conscientious objectors are likely to regard the proposed quota system as an unnecessary constraint on their freedom to object. It does entail the imposition of some form of monitoring for conscientious objectors, which could be perceived as onerous and intrusive (and perhaps they would be). The quota system also applies restrictions on who can object. Compared to the current freedom to object in many jurisdictions, it is obvious that such changes will be regarded as undesirable. We believe, however, that they are a necessary cost of addressing the concerns of opponents to conscientious objection, and form part of a solution that will preserve the right to object in countries where high rates of conscientious objection are impacting service provision. It is certainly preferable to the risk of conscientious objection provisions being removed.

## Religious Discrimination

A quota system for doctors—or other healthcare professionals—that hold beliefs that lead them to oppose elective abortion could be accused of being discriminatory by infringing on their freedom to hold beliefs deeply rooted in their religious tradition.^6^ For example, the Catholic Church opposes abortion in all circumstances, and it is likely that a significant proportion of Catholic doctors will also, as is the case in Italy. Similarly, in Sunni and Shiite Islam, abortion before 4-months gestation is permissible under certain circumstances and after that to save the life of the mother [27].

There does seem to be a correlation between healthcare professionals and students with religious beliefs and an increased likelihood of conscientious objection to practices such as abortion [11, 12, 46, 56, 58]. One study of practicing obstetrician–gynaecologists in the United States showed that those with high religious motivation were the least likely to provide abortion services [57]. Christopher Kaczor notes, however, that even though ‘religion is indeed connected in various ways to the abortion debate … the links between abortion and religion are not simple and straightforward’ [35]. For instance, adherents of the same religion can often be found on different sides of the abortion debate. Kaczor also observes that there are also numerous atheists who are opposed to abortion, and so it is not clear that claims of religious discrimination are sustainable, even if those with certain religious beliefs are disproportionally affected.

Ideally, the initial introduction of quotas should aim to minimise disruption. Quotas should initially be set as high as possible to avoid communicating that those with a conscientious objection are unwelcome in the speciality, whilst also reducing the likelihood that access to abortion services will be compromised. In any case, we have suggested that if a quota system is *not* introduced in some contexts, it is a realistic possibility that healthcare professionals' right to conscientious objection could become increasingly restricted or removed altogether. Moreover, if a quota was introduced in a country that previously did not permit CO, the likely beneficiaries of this will disproportionately be those with religious beliefs.

## Belief Changes

It is possible that some trainees or even consultants may change their views during their careers regarding abortion. For example, they might decide that they no longer have any objection to performing abortions. We assume that the number of individuals that will change their beliefs in either direction once they are already training or working would be small and unlikely to have a significant impact on service provision. A quota system would obviously not be able to account for changes in individual beliefs—its purpose would be to preserve the right to conscientiously object to abortion; ensure that a sufficient number of trained healthcare professionals are available to provide legal abortion services; and to minimise any curtailing of access to legal abortion services.^7^

## Long-Term Effects

Where legal abortion access is demonstrably affected by a high prevalence of conscientious objection, the proposed quota scheme is intended to operate over the long-term—it will take time for its effects to manifest fully. This is not necessarily problematic for opponents of conscientious objection, however—where conscientious objection is legally permitted, a long-term scheme that addresses their concerns regarding service provision would still likely be a significant improvement.

## Geographic Disparities

Having a certain percentage of the healthcare workforce being permitted to conscientiously object may not be sufficient to alleviate shortages of medical professionals willing to provide services in certain geographic regions. For example, it might be that in a particular rural region there are too many healthcare professionals registered as conscientious objectors, and so abortion provision could still be affected. One possible solution would be to limit the percentage of places available in certain hospitals or regions for healthcare professionals who have a conscientious objection. Of course, this may also limit employment options for conscientious objectors to a degree, but again, we regard this as a necessary cost of ensuring that conscientious objection remains available where it is impacting service provision or is introduced to a country that did not previously permit it. For instance, not being able to work in a particular hospital or region is far less onerous than not being able to practice at all.

## Conclusion

As defenders of conscientious objection in healthcare, our primary goal is to preserve the right to conscientiously object in countries where it is under threat. A secondary objective is to demonstrate that there is a cogent pathway for its introduction in countries where conscientious objection is currently not permitted. The proposal we outline—quotas for specialty training—directly addresses the concerns of critics who argue that the right to conscientious objection threatens the provision of legal abortion services. By providing a practicable solution to this issue—one of genuine concern in countries such as Italy—quotas undermine the primary argument for the removal of conscientious objection. Of course, these concerns are also a significant barrier to the introduction of conscientious objection in countries currently without this right. By addressing them via our proposal for quotas and outlining the benefits of conscientious objection, we have provided a strong case for its adoption.

Supporters of conscientious objection may judge this proposal to be a radical one that reduces conscientious objection rights they currently enjoy. However, countries where conscientious objection has no discernible impact on the provision of abortion services are not the target of our proposal—it is aimed at countries where the removal of conscientious objection is a serious threat, as well as countries where the right to object is not currently permitted. Critics of conscientious objection who would prefer that it be removed altogether may also find our proposal unsatisfactory. However, given that it addresses their primary concerns regarding provision of services, and may even help address staff shortages in certain areas, there seems little reason for critics to oppose it.

## Footnotes

1 Whether or not this could be expanded to other healthcare professions or specialties is outside of the scope of this essay. In principle, providing there is strong enough evidence that patients' access to a legal service is being compromised, then the wider use of quotas to ensure access may be indicated.

2 Nevertheless, the relationship between conscientious objection and abortion remains complex, and the limitations of the study by Autorino et al. [1] show that the picture remains incomplete.

3 This has been shown to negatively affect the health of mothers [2].

4 An obvious candidate to meet this criterion would be the practice of non-therapeutic infant male circumcision. Despite being legal, many bioethicists are now challenging the assumption that it is acceptable for male infants to have parts of their sexual organs removed before they are able to understand the consequences of doing so [14].

5 Other candidates would be for specialties and professions involved with euthanasia or physician assisted suicide.

6 For instance, in the Christian tradition, the practice of abortion is described as morally licit as early as the late first century and early second century in the Didache and the Epistle of Barnabas [24].

7 It could be objected that by advocating a quota system we become more complicit in the provision of abortion. However, unless the implementation of a quota system leads to an increase in the number of abortions (which is not self-evident), it is not clear that this would make us (or anyone else) any more complicit than we already are as participants in a democratic society that legally permits abortion. Moreover, our belief is that the system we have proposed will serve to prevent conscientious objection rights from being completely removed in countries where it is clearly affecting service provision, and enable conscientious objection to be introduced in countries which currently do not permit it.
